# Severe anaphylactic shock suspectedly induced by injection of recombinant humanized type III collagen lyophilized fiber for facial skin improvement: a case report

**DOI:** 10.3389/falgy.2026.1901705

**Published:** 2026-09-04

**Authors:** Junjie Liu, Wenjing Yan, Jing Liu, Bowen Yang, Yong Du

**Affiliations:** 1 Department of Plastic Surgery, Yixing Traditional Chinese Medicine Hospital, Yixing, China; 2 Shenzhen University, Shenzhen, China; 3 Department of Plastic Surgery, Jiangnan University Medical Center, Wuxi, China; 4 Department of Plastic Surgery, Wuxi Xinwu District Hongshan Hospital, Wuxi, China; 5 Department of Plastic Surgery, General Hospital of Shenzhen University, Shenzhen, China

**Keywords:** anaphylactic shock, case report, cosmetic injection, immediate-type hypersensitivity, recombinant humanized type III collagen

## Abstract

Recombinant humanized type III collagen is widely used in facial rejuvenation due to its excellent biocompatibility and overall favorable safety profile; however, severe systemic hypersensitivity reactions are exceedingly rare. We report a case of a 47-year-old woman with no prior history of allergy who received topical lidocaine to the face, followed by injection of recombinant humanized type III collagen lyophilized fibers for skin texture improvement. Immediately after the injection, she developed suspectedly severe anaphylactic shock temporally associated with the recombinant collagen injectionpresenting with confusion, respiratory distress, followed by marked bradycardia (33 beats/min), and loss of palpable arterial pulse. Emergency protocols were activated immediately, including cardiopulmonary resuscitation, intravenous adrenaline, corticosteroids, and fluid resuscitation. The patient’s vital signs gradually recovered, and she was discharged after complete recovery two days later after complete recovery. This case suggests that recombinant humanized collagen, as a macromolecular protein, inherently carries the risk of triggering immediate-type anaphylactic shock despite its favorable safety profile. Clinicians should perform rigorous preoperative assessment, adhere to standardized procedures, and be proficient in the emergency management of anaphylactic shock to minimize the risk of severe adverse reactions and ensure medical safety.

**Table 1:** Timeline of key events, interventions, and findings following intradermal collagen injection.

Phase	Time	Key events&immediate actions	Vital signs&examination	Laboratory&imaging
Exposure & early symptoms	15:50–16:02	Facial intradermal injection of recombinant humanized type III collagen; throat discomfort reported → final 3 injections completed	—	—
Deterioration	16:05–16:08	Palpitations → unresponsive when assisted to sit up; placed supine with O₂	BP 115/73 mmHg, HR 70 bpm	—
Cardiac arrest & resuscitation	16:10–16:15	Suspected allergic reaction; IM dexamethasone 5 mg without improvement → PEA cardiac arrest (HR 33 bpm, diaphoresis, pulseless) → immediate CPR + IV epinephrine 1 mg → ROSC	ROSC: BP 113/65 mmHg, HR 66 bpm	—
Immediate post-resuscitation	16:18–16:22	Regained consciousness, cool/clammy skin; IV promethazine 25 mg, dexamethasone 10 mg, epinephrine 2 mg, norepinephrine 2 mg, aggressive fluids	SpO₂ 92%; BP and pulse stabilized; perioral swelling, diffuse erythema, confusion	—
Further stabilization	Resuscitation room	IV dexamethasone 5 mg, hydrocortisone 40 mg; fluid and electrolyte replacement	Persistent shock signs	Lactate 5.19 mmol/L, glucose 10.02 mmol/L, lymphocytes 4.38 × 10⁹/L, eGFR 77 mL/min, total CO₂ 13.4 mmol/L Chest CT: no airway obstruction or pulmonary infection
Outcome	Day 2	No chest tightness, dyspnea; fully alert and oriented	BP, HR, SpO₂ normalized	Clinically stable, discharged

PEA, pulseless electrical activity; ROSC, return of spontaneous circulation; CPR, cardiopulmonary resuscitation; BP, blood pressure; HR, heart rate; SpO₂, peripheral oxygen saturation; IM, intramuscular; IV, intravenous; eGFR, estimated glomerular filtration rate; CT, computed tomography.

## 1 Introduction

The use of injectable dermal fillers to improve cutaneous dryness represents a popular cosmetic procedure. Collagen-based products rank among the most prevalent options, with a compound annual growth rate (CAGR) of 31.3%, and robust market growth is projected to continue. Key factors contributing to this popularity include procedural ease, minimal recovery time, and a low incidence of side effects. Although routine pre-procedural skin testing is not currently required for collagen-based products in clinical practice, these products still possess inherent allergenicity as macromolecular proteins. While such adverse events are mostly mild, they can occasionally lead to anaphylactic shock—a condition characterized by abrupt onset, rapid progression, and high mortality. Hypersensitivity reactions to injectable materials—sometimes mislabeled as “inflammatory reactions”—are rare, and there is a paucity of documented allergic reactions to filler products in the literature.

Herein, we present a case of anaphylactic shock following the intradermal injection of recombinant humanized type III collagen. Through retrospective analysis of its clinical characteristics, emergency management, and prognosis, we aim to explore the underlying pathogenesis, potential sensitizing factors, and critical points in clinical prevention. This report intends to heighten clinical awareness of these sporadic but severe adverse events and to provide a reference framework for the prevention and management of severe hypersensitivity reactions.

## 2 Case report

A 47-year-old woman with an unremarkable past medical history—no known food or drug allergies, no chronic diseases, no family history of hereditary disorders, and no prior surgeries— received intradermal injections of recombinant humanized type III collagen for the treatment of facial dryness. Before the procedure, topical lidocaine cream was applied to the face for 40 min as surface anesthesia. The facial skin was then disinfected three times with povidone-iodine solution and rinsed with sterile normal saline. The investigation product, lyophilized recombinant humanized type III collagen (manufacturer: Changsha Juyuan; composition: recombinant humanized type III collagen only; sterile by irradiation; 24-month shelf life at 10–30 °C), was reconstituted and uniformly injected into the dermis of the face at a depth of approximately 1.3 mm. The injection was started at 15:50 on January 15, 2024. At 16:02, the patient reported throat discomfort; accidental ingestion of the anesthetic was considered, and the final three injections were completed. At 16:05, she reported palpitations and was placed in the supine position and given supplemental oxygen. At 16:08, after being helped to sit up, she became unresponsive. Blood pressure was 115/73 mmHg and the pulse was 70 bpm. At 16:10, an allergic reaction was suspected; intramuscular dexamethasone 5 mg was given without improvement, and the emergency team was called. At 16:15, the emergency physician found no carotid pulse, inaudible heart sounds, slightly dilated pupils, and diaphoresis. The monitor showed a heart rate of 33 bpm with pulseless electrical activity. Cardiopulmonary resuscitation was started immediately, intravenous adrenaline 1 mg was administered, and intravenous access was secured. After return of spontaneous circulation, her blood pressure was 113/65 mmHg and pulse 66 bpm. At 16:18, she regained consciousness but had cool, clammy skin and an SpO₂ of 92%. She was transferred to the resuscitation room at 16:22. The initial working diagnosis was anaphylactic shock suspected to be secondary to the recombinant type III collagen injection, although concurrent exposures to lidocaine and povidone-iodine could not be excluded. Treatment in the resuscitation room consisted of placing the patient in the supine position with supplemental oxygen, continuous cardiac monitoring, and administering intravenous promethazine 25 mg, dexamethasone 10 mg. Epinephrine and norepinephrine were administered as continuous intravenous infusions after return of spontaneous circulation (ROSC), together with aggressive intravenous fluid resuscitation to stabilize blood pressure and pulse. With these interventions, the patient gradually regained full consciousness and became responsive; however, signs of shock persisted. Further laboratory investigations showed elevated lactate (5.19 mmol/L), blood glucose (10.02 mmol/L), and lymphocyte count (4.38 × 10⁹/L), along with a reduced estimated glomerular filtration rate (77 mL/min) and low total CO₂ (13. 4 mmol/L). On physical examination, the patient was confused, with perioral swelling and diffuse generalized erythema. In response to these findings, intravenous fluid and electrolyte replacement was administered to maintain homeostasis, along with corticosteroid therapy: intravenous dexamethasone 5 mg and hydrocortisone 40 mg. A chest computed tomography scan showed no evidence of airway obstruction or pulmonary infection. After two days of treatment, the patient was free of chest tightness and dyspnea, or other discomfort; she was fully alert and oriented, and repeat vital signs confirmed normalization of blood pressure, heart rate, and oxygen saturation. She was discharged in a clinically stable condition. The specific timeline is shown in Table 1.

The patient did not return to our hospital for the scheduled follow-up. One week after discharge, she independently presented to another hospital for allergen-specific IgE testing, which revealed a cow's milk allergy.

## 3 Discussion

### 3.1 Product characteristics and adverse reaction profile

Recombinant humanized type III collagen is a novel biomedical material constructed by optimizing the original gene sequence of human skin type III collagen. It possesses excellent water solubility, high bioactivity, good biocompatibility, and low immunogenicity. Moreover, it can be progressively degraded and absorbed by human collagenases (1), demonstrating performance far superior to that of native human collagen. As one of the earliest injectable fillers introduced into clinical practice over 30 years ago, collagen can effectively improve skin elasticity, enhance hydration, and reduce wrinkles, thereby occupying a core role in skin rejuvenation. Because collagen promotes cellular integration into the host tissue, it is cleared relatively slowly from the injection site. Consequently, should an allergic reaction develop, management is largely limited to targeted symptomatic therapy, as the material cannot be rapidly eliminated. The recombinant type III collagen used in the present case is engineered by optimizing the human collagen gene sequence, which confers superior biocompatibility, bioactivity, safety, and cell adhesion properties (2). Beyond directly filling depressed areas of the skin, this material provides an optimal microenvironment for fibroblasts, thereby stimulating extracellular matrix (ECM) synthesis and attenuating the degradation of collagen and elastic fibers. These effects translate into reduced transepidermal water loss (TEWL) and diminished hyperpigmentation, resulting in a healthier and more even skin appearance (3). Although recombinant collagen generally exhibits a favorable safety profile, complications may still arise and are typically categorized as minor or severe. Minor complications include transient swelling, mild erythema, nodule formation, pain, and mild allergic reactions, most of which usually resolve within 24 to 48 h. Severe complications encompass irreversible tissue damage caused by vascular embolism, such as skin necrosis and visual impairment (4), as well as IgE-mediated anaphylactic shock. These adverse events are characterized by an acute onset and rapid progression and pose a life-threatening risk to the patient. Therefore, they warrant an exceptionally high level of vigilance among clinicians.

### 3.2 Pathogenesis of anaphylactic shock

The core mechanism of immediate-type anaphylactic shock primarily involves immunoglobulin E (IgE)-mediated type I hypersensitivity. In brief, when collagen acts as an antigen and enters the body, it activates helper T cells, to secrete cytokines such as IL-2, IFN-*γ*, and TNF, which drive B cells to produce allergen-specific IgE. These IgE antibodies bind to the high-affinity receptor Fc*ε*RI on the surface of mast cells and basophils. Upon re-exposure to the identical allergen, antigen-antibody cross-linking occurs, inducing extensive Fc*ε*RI aggregation and initiating a cascade of intracellular signaling events. This culminates in the explosive release of abundant preformed mediators, including histamine, leukotrienes, tryptase, and other inflammatory mediators within minutes. In parallel, mast cells generate lipid mediators through the arachidonic acid pathway, which are liberated into the circulation following IgE cross-linking. Collectively, these mediators cause vasodilation, mucosal edema, bronchoconstriction, and hypotension (5).

Recombinant type III collagen, as a macromolecular protein, theoretically possesses the potential to function as a complete antigen or a hapten carrier. In the present case, the extremely short interval from injection to the onset of laryngeal discomfort and loss of consciousness is consistent with the typical temporal sequence of type I hypersensitivity. Notably, allergen screening after recovery revealed only elevated milk-specific IgE, while collagen-specific IgE remained undetectable. To account for this observation, we speculate that this process may be related to the “IgE threshold effect” (6): owing to individual variations in mast cell surface Fc*ε*RI density and intracellular signaling thresholds, even low levels of allergen-specific IgE that fall below conventional assay cutoffs can trigger mediator release if a critical cross-linking threshold is exceeded. The concomitant presence of milk sensitization in this patient may further suggest a generally lower mast cell activation threshold. Thus, it is conceivable that low-level collagen sensitization, although undetectable by standard assays, was sufficient to provoke a hypersensitivity reaction. However, this interpretation remains speculative, and the precise mechanism awaits further investigation.

### 3.3 Diagnosis and management principles of anaphylaxis

#### 3.3.1 Diagnosis and differential diagnosis

According to the 2023 updated Joint Task Force on Practice Parameters (JTFPP) (7) and the World Allergy Organization (WAO) 2020 guidelines (8), the diagnosis of anaphylaxis relies predominantly on the acute onset of clinical symptoms. The primary diagnostic pathways and their components are as follows (Table 2):

**Table 2:** Diagnostic pathways for acute allergic reactions.

Pathway	Basis for diagnosis
Pathway 1 (Skin/mucosal involvement required)	Acute onset within minutes to hours, with involvement of the skin and/or mucosa, and at least one of the following: a. Respiratory compromise [e.g., dyspnea, wheezing, bronchospasm, stridor, reduced peak expiratory flow (PEF), hypoxemia]. b. Circulatory compromise (e.g., hypotension, hypotonia/collapse, syncope, urinary or fecal incontinence). c. Severe gastrointestinal symptoms (e.g., intense crampy abdominal pain, repetitive vomiting, intestinal colic, defecation urgency), particularly after exposure to non-food allergen.
Pathway 2 (Skin/mucosal involvement NOT required)	Acute onset (within minutes) after exposure to a known or highly probable allergen, presenting with at least one of the following in the absence of typical skin manifestations: a. Hypotension; b. Bronchospasm; c. Laryngeal involvement (e.g., stridor, laryngeal edema).

PEF, peak expiratory flow.

In the present case, the patient developed perioral swelling, generalized skin erythema, respiratory distress, throat discomfort, and urinary incontinence shortly after the injection, thereby fulfilling the above-mentioned diagnostic criteria. The multi-system presentation nonetheless establishes the clinical diagnosis of anaphylactic shock. Although lidocaine and povidone-iodine were also potential triggers, lidocaine is known to induce hypersensitivity reactions: one review of the English-language literature (1950–2011) identified IgE-mediated allergic reactions in 29 of 2,978 patients, yielding an incidence of 0.97% (9)^,^ which is extremely rare, typically presenting as limited urticaria rather than severe shock (10). Meanwhile, systemic anaphylaxis to povidone-iodine is only sporadically reported. After excluding these ancillary agents as probable causes, the recombinant human collagen-based material remains the most plausible trigger, based on the immediate temporal association with the injection. Definitive identification of the culprit allergen would require skin testing with the specific recombinant collagen product and each co-administered substance, which was not performed. Once this diagnosis was established, the main alternative diagnoses were considered and excluded as follows.

Vasovagal syncope was excluded because the patient presented with pruritus and sustained symptoms, and lacked the classic prodromal features of bradycardia, pallor, and diaphoresis (11). Local anesthetic systemic toxicity was also considered unlikely, because this condition typically manifests with perioral numbness, seizures, or ventricular arrhythmias rather than the observed multisystem anaphylactic presentation (12). Although non-IgE-mediated anaphylaxis due to direct mast cell activation or complement-mediated pseudoallergy cannot be fully excluded without basophil activation tests, the patient's prior tolerance and positive milk-specific IgE favor an IgE-mediated pathway (13). These differential diagnostic considerations, together with the temporal relationship described, further support recombinant humanized type III collagen as the likely trigger. Notably, during anaphylactic shock, vasodilation and a reduction in effective circulating blood volume ordinarily trigger compensatory sympathetic activation, which manifests as tachycardia. However, in this case, the patient exhibited a marked decline in heart rate (<33 beats/min) early in the clinical course, and such paradoxical bradycardia frequently portends a critical condition. This phenomenon is currently explained primarily by the Bezold–Jarisch reflex (14): during anaphylactic shock, peripheral vasodilation causes a profound decrease in venous return, and cardiac mechanoreceptors sense the resultant diminished ventricular filling, reflexively inducing bradycardia. Therefore, clinicians should maintain a high index of suspicion for severe anaphylactic shock presenting with bradycardia and be able to promptly recognize this life-threatening presentation.

#### 3.3.2 Management principles

Anaphylactic shock is a severe, systemic hypersensitivity reaction that, if not promptly treated, can swiftly progress to cardiac arrest, culminating in circulatory and respiratory failure. The World Allergy Organization (WAO) 2020 guidelines and the 2020 update from the American Academy of Allergy, Asthma & Immunology (7, 8) state that intramuscular adrenaline is the first-line treatment for anaphylaxis and has no absolute contraindications. Additionally, international guidelines consistently identify antihistamines and glucocorticoids as second-line agents for anaphylaxis (15), which must only be used as adjuncts to adrenaline. However, current evidence shows that both antihistamines and glucocorticoids have a relatively slow onset and lack robust proof of efficacy in the acute phase of anaphylactic shock. They should never replace adrenaline or delay its timely administration (16). Guidelines recommend a rapid infusion of 20 mL/kg crystalloid for hemodynamic instability, and high-flow oxygen to maintain SpO₂ > 95% (17, 18).

The sequence of interventions in this case illustrates the individualized application and dynamic adjustment of these principles. The patient had no palpable arterial pulses and inaudible heart sounds, signifying cardiac arrest. Therefore, the management should follow cardiac arrest resuscitation guidelines—chest compressions combined with an intravenous bolus of 1 mg adrenaline, under continuous electrocardiographic monitoring. Available data indicate that the intravenous route is more likely to provoke adverse cardiovascular events, especially fatal arrhythmia (19). Notably, intravenous adrenaline is therefore indicated exclusively for acute resuscitation in cardiac arrest, rather than for routine use in anaphylactic shock. If shock remains refractory, current guidelines recommend adding a second-line vasopressor to maintain hemodynamic stability, specifically norepinephrine (20). Importantly, all intravenous vasopressors administration must be performed under continuous electrocardiographic monitoring, with strict vital-sign surveillance to prevent malignant arrhythmia.

In summary, the clinical management of anaphylactic shock focuses on three pillars of resuscitation: adrenaline, fluid resuscitation, and airway management. Adjunctive medications such as antihistamines and glucocorticoids can help alleviate allergic symptoms during this process.

### 3.4 Measures to reduce anaphylactic shock induced by injection

Based on an in-depth analysis of the pathogenesis of anaphylactic shock caused by recombinant human type III collagen injection, we discuss strategies to mitigate injection risk from the perspectives of health status, skin condition, individual sensitivity, and preoperative risk assessment (21).

#### 3.4.1 Rigorous patient selection and comprehensive informed consent

Rigorous patient selection constitutes the most critical step in preventing post-injection complications. Prior to the procedure, a complete and detailed allergy history must be obtained (22), so as to exclude potential cross-reactivity. Key elements include: (i) a history of atopic diseases such as allergic rhinitis, asthma, or atopic dermatitis; (ii) previous exposures and any adverse reactions to collagen-containing products; (iii) known hypersensitivity to bovine or porcine collagen, which may indicate cross-reactivity risk; (iv) medication allergies, particularly to antibiotics or local anesthetics that might be used concurrently; (v) any unexplained episodes of urticaria, angioedema, or anaphylactoid symptoms, which could signify an underlying mast cell disorder or heightened basophil reactivity.

Patients should also be evaluated for severe infectious diseases (23), which can alter immune tolerance to macromolecules such as collagen. Especially,the skin condition of the treatment area must be carefully assessed for active infections (e.g.,herpes simplex, folliculitis) and inflammatory lesions (e.g., acne), which heightens the likelihood of an allergic response. On this basis, patients must be candidly informed of the potential risks and provide written informed consent.

#### 3.4.2 Standardized injection procedures, environmental preparedness and post-injection observation

Standardized injection technique represents another essential dimension of risk control. The injector must possess precise knowledge of facial anatomy and select the correct injection plane and technique according to the rheological properties of the product. For example, in highly vascularized regions, blunt cannulas should be preferred over sharp needles to minimize the risk of inadvertent intravascular injection and the secondary hypersensitivity reactions. Strict aseptic technique must be maintained throughout the procedure to prevent infection and inflammation, thereby avoiding the activation of hypersensitivity pathways (24).

Equally critically the injection setting is recommended to be equipped with epinephrine, glucocorticoids, antihistamines, and the supplies necessary for intravenous access. The patient must be closely monitored during the injection. At the first appearance of prodromal signs of anaphylaxis—such as pruritus, urticaria, cyanosis of the lips, laryngeal edema, or a drop in systolic blood pressure—emergency measures should be initiated without delay. Adrenaline should be administered promptly, and once vital signs are stabilized, the patient should be rapidly transferred to a more comprehensively equipped medical facility for further management.

### 3.5 Limitations

This case report has several limitations. First, documentation gaps inherent to emergency settings exist: a detailed psychosocial history and serial neurological and skin examinations were not recorded during acute resuscitation. Second, confirmatory allergy testing for collagen, lidocaine, and povidone-iodine was not performed, and acute serum tryptase was not measured; the exact trigger and immunological mechanism therefore remain unconfirmed. Third, the patient did not attend scheduled follow-up, and post-discharge allergen testing at another facility revealed only milk-specific IgE, which does not establish a causal link to the acute reaction. Fourth, because the drug has not yet been marketed, a clinical trial registration number is not currently available. Additionally, the patient's personal perspective on the event and its psychological impact could not be obtained, as the emergency precluded contemporaneous discussion and follow-up was lost. Despite these limitations, the well-documented acute timeline and the exclusion of alternative diagnoses provide reasonable support for the conclusion that recombinant humanized type III collagen was the probable trigger. Prospective data collection in future cases would help address these gaps.

## 4 Conclusion

In conclusion, this case demonstrates that anaphylactic shock, although rare, can occur in temporal association with recombinant humanized type III collagen injection. The exact causative agent cannot be established with certainty, and the possibility of co-administered lidocaine as a contributor cannot be excluded. Nevertheless, the event highlights the critical need for vigilance during collagen-based aesthetic procedures. Prompt recognition of systemic allergic reactions and immediate intramuscular adrenaline administration, as recommended by current anaphylaxis guidelines, are essential. Adequate emergency preparedness should be ensured to safeguard patient safety.

## Data Availability

The original contributions presented in the study are included in the article/Supplementary Material, further inquiries can be directed to the corresponding author.
